# Systematic review and meta-analysis shows a specific micronutrient profile in people with Down Syndrome: Lower blood calcium, selenium and zinc, higher red blood cell copper and zinc, and higher salivary calcium and sodium

**DOI:** 10.1371/journal.pone.0175437

**Published:** 2017-04-19

**Authors:** Amene Saghazadeh, Maryam Mahmoudi, Atefeh Dehghani Ashkezari, Nooshin Oliaie Rezaie, Nima Rezaei

**Affiliations:** 1 Research Center for Immunodeficiencies, Children's Medical Center, Tehran University of Medical Sciences, Tehran, Iran; 2 MetaCognition Interest Group (MCIG), Universal Scientific Education and Research Network (USERN), Tehran, Iran; 3 Department of Cellular and Molecular Nutrition, School of Nutrition and Dietetics, Tehran University of Medical Sciences, Tehran, Iran; 4 Dietitians and Nutrition Experts Team (DiNET), Universal Scientific Education and Research Network (USERN), Tehran, Iran; 5 NeuroImmunology Research Association (NIRA), Universal Scientific Education and Research Network (USERN), Tehran, Iran; 6 Systematic Review and Meta-analysis Expert Group (SRMEG), Universal Scientific Education and Research Network (USERN), Boston, MA, United States of America; 7 Department of Immunology, School of Medicine, Tehran University of Medical Sciences, Tehran, Iran; TNO, NETHERLANDS

## Abstract

Different metabolic profiles as well as comorbidities are common in people with Down Syndrome (DS). Therefore it is relevant to know whether micronutrient levels in people with DS are also different. This systematic review was designed to review the literature on micronutrient levels in people with DS compared to age and sex-matched controls without DS. We identified sixty nine studies from January 1967 to April 2016 through main electronic medical databases PubMed, Scopus, and Web of knowledge. We carried out meta-analysis of the data on four essential trace elements (Cu, Fe, Se, and Zn), six minerals (Ca, Cl, K, Mg, Na, and P), and five vitamins (vitamin A, B9, B12, D, and E). People with DS showed lower blood levels of Ca (standard mean difference (SMD) = −0.63; 95% confidence interval (CI): −1.16 to −0.09), Se (SMD = -0.99; 95% CI: -1.55 to -0.43), and Zn (SMD = -1.30; 95% CI: -1.75 to -0.84), while red cell levels of Zn (SMD = 1.88; 95% CI: 0.48 to 3.28) and Cu (SMD = 2.77; 95% CI: 1.96 to 3.57) were higher. They had also higher salivary levels of Ca (SMD = 0.85; 95% CI: 0.38 to 1.33) and Na (SMD = 1.04; 95% CI: 0.39 to 1.69). Our findings that micronutrient levels are different in people with DS raise the question whether these differences are related to the different metabolic profiles, the common comorbidities or merely reflect DS.

## Introduction

Down Syndrome (DS) or trisomy 21 is a congenital condition characterized by phenotypic features as well decreased growth and development. The major maternal risk factors are advanced age [1] and impaired folate-homocysteine metabolism [2]. Pregnant women can be screened if they carry a fetus with DS [3]. If these results are out of line, doctors can confirm prenatal diagnosis [4, 5]. Globally, most confirmed pregnancies are terminated; average DS pregnancy termination rates are 67% and 85% [6]. Nevertheless, DS remains the most common recognized genetic cause of mental retardation and is reported to affect approximately 1 in 732 life born American infants (∼0.14%) [7]. Similar levels are found in the Netherlands (between 0.14 and 0.15%) [8]. Because of a growing trend in advanced maternal age, the frequency of DS has more than doubled over recent decades [9]. Moreover, the prevalence of this lifetime condition is increasing as the life-expectancy of people with DS has increased to 60 years [10].

DS is associated with various life-limiting or life-threatening comorbidities. Congenital heart disease is the most common cause of death at adulthood, pneumonia and other respiratory infections at both childhood and senescence [9]. In addition, people with DS frequently suffer from other complications affecting their quality of life (QoL). They suffer from different degrees of cognitive impairment that may hamper their memory function [11] and neurodevelopmental disorders, such as autism spectrum disorders. Early-onset neurological diseases like dementia and seizure, are relatively common [12, 13]. Resting metabolic rate is reduced in people with DS, making them more prone to develop metabolic disorders, as overweight, obesity, and diabetes [14–16]. Immune-mediated disorders such as celiac disease and thyroid disorders (hypo- or hyperthyroidism, and autoimmune thyroiditis), also affect people with DS more frequently [17]. Due to the high rates of comorbidity, specific clinical guidelines are developed to manage health and quality of life of people with DS (4).

Besides these clinical condition, several metabolic profiles are different in people with DS: the amino acid profile (low serotonin [18, 19] and serine [20], high lysine [21] and cysteine [20] in blood), low gamma-Aminobutyric acid and glutamate levels in the central nervous system [22]. Also, hormonal changes occur, most notably thyroid dysfunction (low T-4 and high TSH) and gonadal dysfunction (high FSH and LH) [23, 24]. Despite these observations, clinical studies have not provided evidence that normalization of amino acid or thyroid hormone profile improves health, growth, or QoL [23, 25].

Micronutrients perform complex metabolic functions to preserve metabolic balance [26]: Fe and the trace elements Zn, Cu and Se act as coenzymes, while vitamins A, C and E, act as free radical scavengers. Their deficiency or overload may contribute to cell injury. Since high prevalence of comorbidities and differences in metabolic profiles exist, we undertake the current study to evaluate whether micronutrient levels in people with DS are different. Therefore we conduct a systematic review and meta-analysis study on the micronutrient status in people with DS.

## Materials and methods

We use the preferred reporting items for systematic reviews and meta-analyses (PRISMA) statement [27] to improve the present systematic review and meta-analysis (S1 PRISMA Checklist and S1 Fig). Prior the authors (AS and NR) developed the study protocol which is available on request.

### Literature search and meta-analysis

We conducted the present systematic review and meta-analysis to recognize all studies measuring concentrations of five trace elements (Cu, Fe, Mn, Se, and Zn), six minerals (Ca, Cl, K, Mg, Na, and P), and six vitamins (vitamins A, B9, B12, C, D, and E) in whole blood, red cells, plasma, serum, hair or saliva among people with DS and simultaneously in age, sex, and race matched healthy controls. We identified relevant studies from January 1967 to April 2016 by searching electronic medical databases, PubMed, Scopus, and Web of knowledge (S1 Text). To find additional studies, we also checked reference lists of all relevant articles.

Original articles were included if they met both criteria; 1) they did measure levels of micronutrients in the samples we study (whole blood, plasma, serum, red cells, hair and saliva) in people with DS and healthy controls, and 2) provided results, including the total number of subjects and controls with mean and standard deviation (SD). We also included studies providing sufficient data (such as median, first quartile, and third quartile, or median and range, or median and standard error) to calculate mean and SD. We excluded studies that measured protein or mRNA expression of micronutrients in tissues or samples other than red cells.

We extracted from each included publication; first-named author, year of publication, location of study, the assay that was used for micronutrient measurement, type of specimen taken from subjects, number of subjects and controls, demographic characteristics, mean ± SD of the micronutrient levels and the used scale of micronutrient levels.

We performed all of the statistical analyses using Review Manager Version (Version 5.3. Copenhagen: The Nordic Cochrane Centre, the Cochrane Collaboration, 2014). As explained elsewhere [28], we created the continuous type of outcome and entered the number of participants in subject and control groups and the mean and SD of the micronutrient levels. Fixed effects and random effects were interchangeably used as the analysis model. Heterogeneity was determined using Q statistic tests and the I^2^ index. According to the Cochrane guidelines, an I^2^ less than 40% would mean that the inconsistency across studies is not important. In this case, we planned to use the fixed effects model. If the I^2^ estimates fluctuated more than 40% we intended to use the random effects procedure as the analysis model. As well, the standardized mean difference (SMD) and mean difference (MD) were interchangeably used for measurement of effect. The SMD was applied if studies used different measurement scales or assays. Otherwise we used the MD for measurement of effect. Publication bias was assessed when there were five or more than five studies using the degree of funnel plot asymmetry. A *P* value less than 0.05 was considered significant.

### Study selection and data extraction

**Fig 1:**
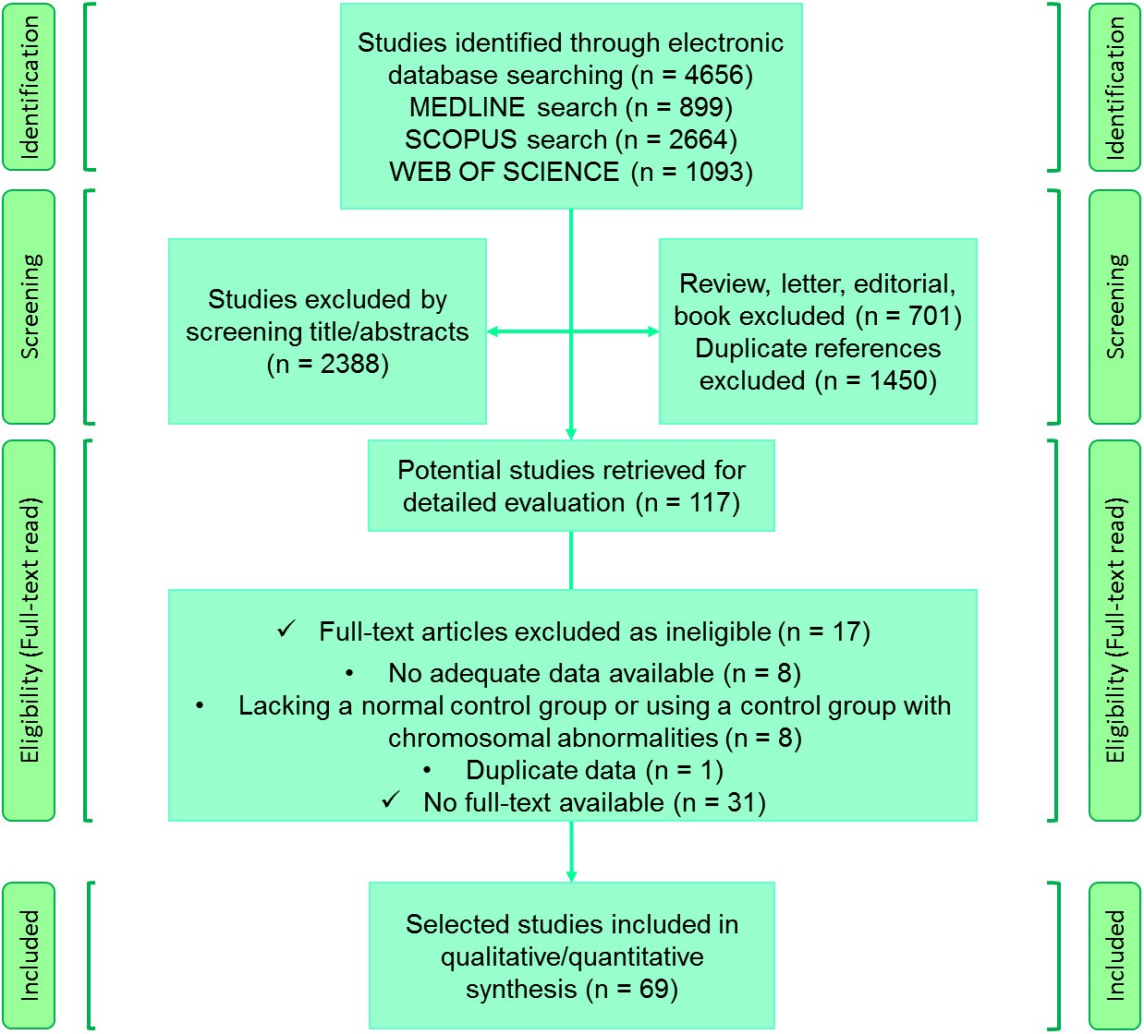
Search results and study selection.

As recommended by the PRISMA guidelines and graphically illustrated in Fig 1, the study selection is a procedure composed of four main steps: identification, screening, eligibility, and inclusion. The “identification” step aimed at acquisition of all the relevant papers is a process including forward and backward searches and then removal of duplicate records. The “screening” step is to screen results based on title and/or abstract. The apparently relevant papers are examined by the authors for “eligibility”. The final step is to include articles that met eligible criteria in systematic review and in meta-analysis if applicable. The initial search resulted in 4,656 records (Fig 1). After removing duplicate publications (n = 1,450) and excluding reviews, letters, editorials, or book chapters (n = 701), 2,505 discrete manuscripts were identified for review. Of these, 2,388 publications were excluded based on title and/or abstract. We reviewed the remaining 117 publications. Based on detail review, we excluded 40 additional publications: eight articles were excluded because they did not report adequate data [29–36] another eight because a healthy control group lacked [37–44]. One was a duplicate record [45]. Some abstracts or titles were likely to be related, but the full texts were not available to obtain sufficient data for analysis or to ensure they were relevant [46–76]. Eventually, sixty nine studies were included [77–145]. Meta-analysis was performed when there were three or more comparisons regarding the title. Thus we could not carry out quantitative synthesis when there were less than three comparisons. Characteristics of included studies are summarized in S1, S2 and S3 Tables.

### Quality assessment

We appraised the quality of included studies using the Newcastle–Ottawa Scale (NOS) designed for observational studies [146]. The NOS is composed for the assessment of three main aspects of observational studies; sample selection, comparability of cases and controls, and exposure. Using this scale, possible scores range from 0 to 9. Studies with scores of 7–9 stars have the lowest risk of bias and represent the highest quality, whereas studies with scores less than 4 stars have the highest risk of bias and the lowest quality. Studies with scores of 4–6 stars have the moderate risk of bias and quality.

## Results

**Fig 2:**
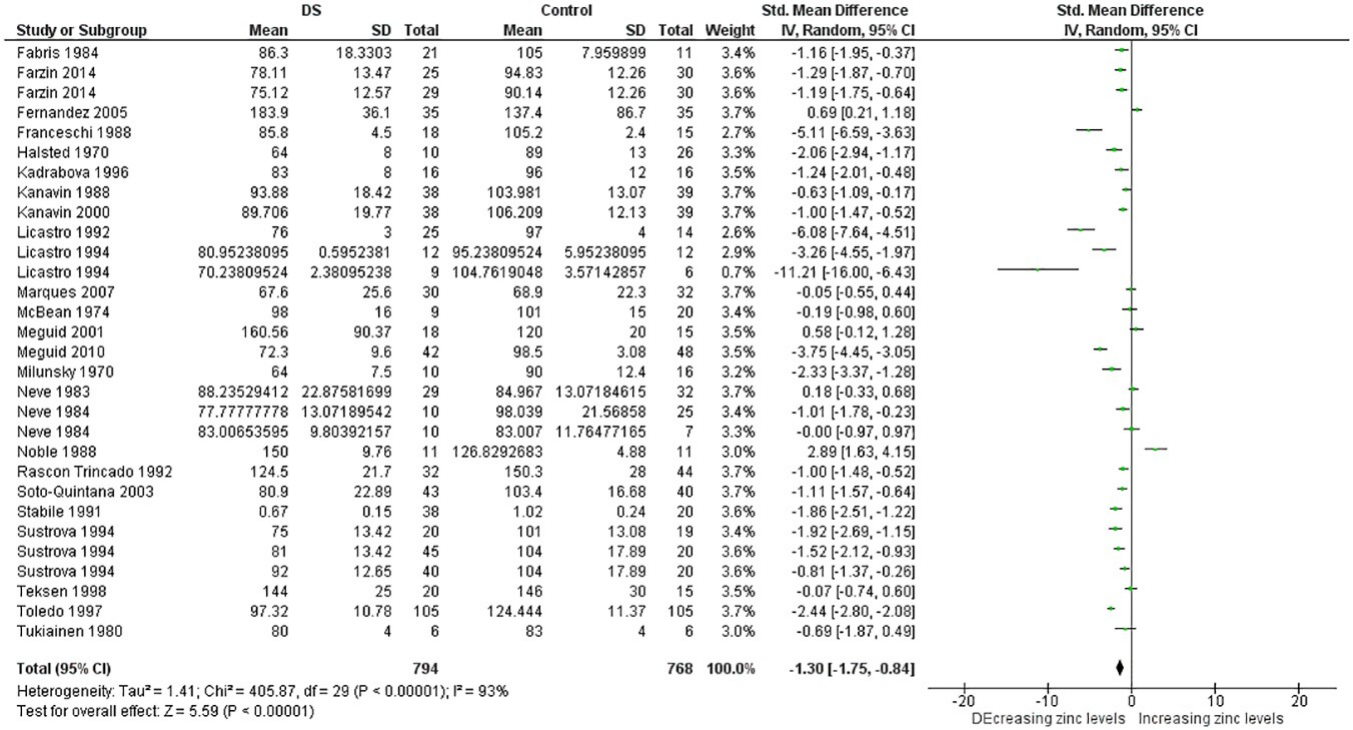
Meta-analysis of blood levels of zinc.

**Fig 3:**
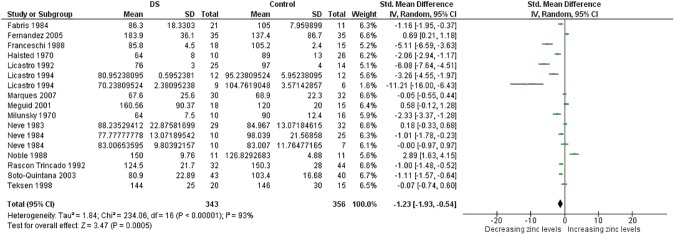
Meta-analysis of plasma levels of zinc.

**Fig 4:**
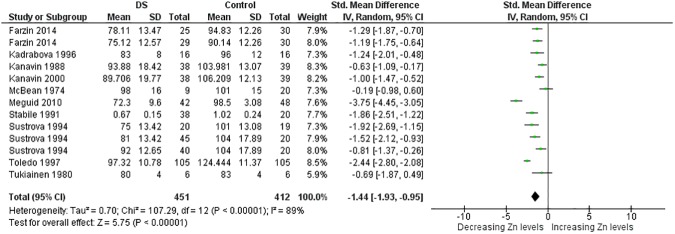
Meta-analysis of serum levels of zinc.

**Fig 5:**
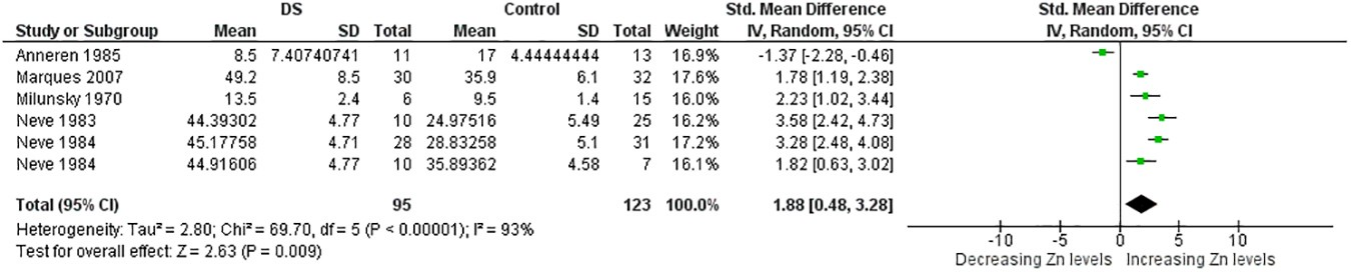
Meta-analysis of intra-erythrocyte levels of zinc.

**Fig 6:**
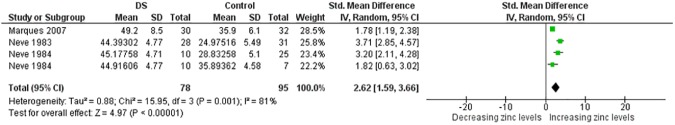
Meta-analysis of intra-erythrocyte levels of zinc.

**Fig 7:**
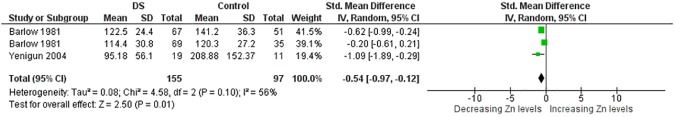
Meta-analysis of hair levels of zinc.

**Fig 8:**
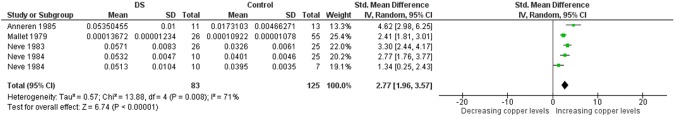
Meta-analysis of intra-erythrocyte levels of copper.

**Fig 9:**
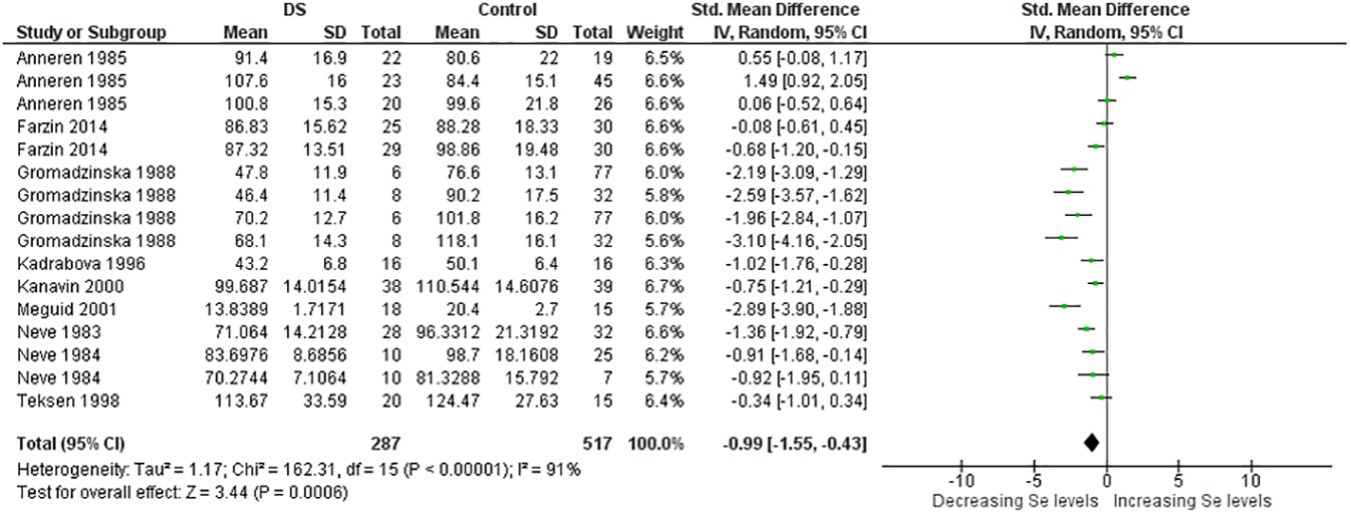
Meta-analysis of blood levels of selenium.

**Fig 10:**
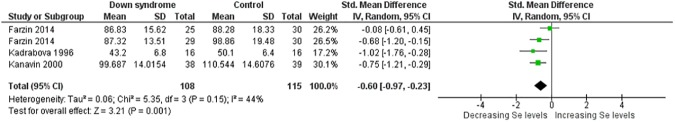
Meta-analysis of serum levels of selenium.

**Fig 11:**
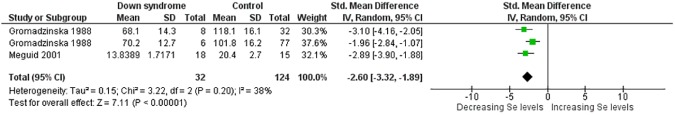
Meta-analysis of whole blood levels of selenium.

**Fig 12:**
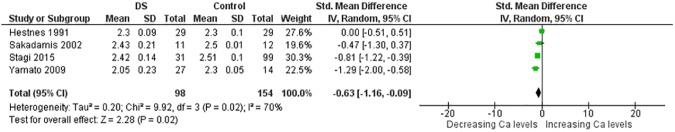
Meta-analysis of blood levels of calcium.

**Fig 13:**
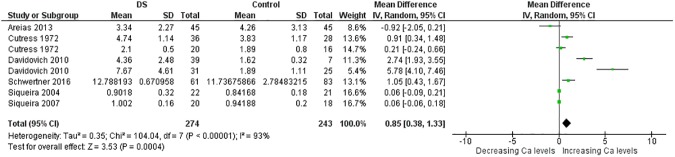
Meta-analysis of salivary levels of calcium.

**Fig 14:**
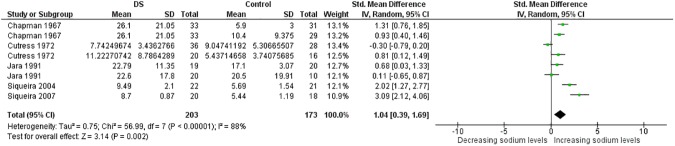
Meta-analysis of salivary levels of sodium.

More than thirty meta-analyses were performed and the S4 Table provides an overview of all these meta-analyses and relevant findings. Here, due to limitations of space, the results of meta-analyses associated with significant p value are expressed in Table 1. Significant results were obtained for the trace elements Cu, Se and Zn and for the minerals Ca and Na too (Figs 2–14). However the most striking results were related to the trace element Zn. Thirty one studies were retrieved on Zn measurements [77–107]. They were published between 1970 and 2014 and all but four conducted in Europe [81–88, 90–98, 102] or America. The largest analysis was performed on thirty comparisons including 1,562 participants and indicated lower blood levels of Zn in people with DS than in control subjects. Also plasma, serum, and red blood cell Zn values were lower. But hair Zn levels were higher in people with DS. Similarly, a meta-analysis of sixteen comparisons involving 804 participants revealed lower blood Se concentrations in people with DS. Additionally, blood Ca was decreased. But salivary levels of Ca and Na were increased (Table 1).

**Table 1:** Summary of meta-analyses associated with the significant *p* value.

Outcome	Comparisons (n)	Cases (n) Controls (n)	Heterogeneity chi^2^ *p* value	Inconsistency I^2^%	Effect measure SMD 95% CI	Overall effect Z *p* value
Blood Zn	30	794/768	405.87 (< .00001)	93	−1.30 [−1.75, −0.84]	5.59 (< .00001)
Plasma Zn	17	343/356	234.06 (0.0005)	93	−1.23 [−1.93, −0.54]	3.47 (0.0005)
Serum Zn	13	451/412	107.29 (< .00001)	89	−1.44 [−1.93, −0.95]	5.75 (< .00001)
RBC Zn	6	95/123	69.70 (< .00001)	93	1.88 [0.48, 3.28]	2.63 (0.009)
RBC Zn	4	78/95	15.95 (0.001)	81	2.62 [1.59, 3.66]	4.97 (< .00001)
Hair Zn	3	155/97	4.58 (0.1)	56	−0.54 [−0.97, −0.12]	2.50 (0.01)
RBC Cu	5	83/125	13.88 (0.008)	71	2.77 [1.96, 3.57]	6.74 (< .00001)
Blood Se	16	287/517	162.31 (< .00001)	91	−0.99 [−1.55, −0.43]	3.44 (0.0006)
Serum Se	4	108/115	5.35 (0.15)	44	−0.60 [−0.97, −0.23]	3.21 (0.001)
Whole blood Se	3	32/124	3.22 (0.20)	38	−2.60 [−3.32, −1.89]	7.11 (< .00001)
Blood Ca	4	98/154	9.92 (0.02)	70	−0.63 [−1.16, −0.09]	2.28 (0.02)
Saliva Ca	8	274/243	104.04 (< .00001)	93	0.85 [0.38, 1.33]	3.53 (0.0004)
Saliva Na	8	203/173	56.99 (< .00001)	88	1.04 [0.39, 1.69]	3.14 (0.002)

## Discussion

The present systematic review was designed to review current literature on micronutrient levels in people with DS compared with controls. We identified sixty nine studies by electronic medical databases up to April 2016. Meta-analysis was performed if there were three or more comparisons regarding the title. Accordingly we were able to carry out meta-analysis of data regarding four trace elements (Cu, Fe, Se, and Zn), six minerals (Ca, Cl, K, Mg, Na, and P), and four vitamins (vitamin A, B9, B12, D, and E). As noted in Table 1, people with DS had lower blood levels for Zn, Se, and Ca and higher red blood cell levels for Cu and Zn (Figs 2–14). Also, lower hair levels of Zn and higher salivary levels of Ca and Na were found. No differences were found between cases and controls with regard to Cl, Fe, K, Mg, P, and vitamin levels.

We found evidence that micronutrient status is different in people with DS for the trace elements Cu, Se, and Zn and also for the minerals Ca and Na. Amino acid abnormalities and high parathyroid hormone (PTH) levels may be implicated in micronutrient changes in people with DS. The important consequences (including thyroid dysfunction, immune disorders, and growth abnormalities) that will follow from this condition are among the most common comorbidities in people with DS.

### Causes

#### Amino acid abnormalities

Analysis of amniotic fluid indicated elevations in essential amino acid levels in the DS group compared to the healthy group. This might reflect profound amino acid deficiency in fetuses with DS as demonstrated in cortical tissues [147]. Deficiency of essential amino acid persists in the elderly people with DS [148]. Therefore, people with DS display altered amino acid profile from gestation throughout lifetime. Abnormal amino acid metabolism might predispose individuals to serious health problems, importantly brain and behavior disorders. This might explain why dementia occurs more frequently and earlier in people with DS [149]. Additionally amino acids and their binding to trace elements (notably Zn) help maintain proper trace elements levels [150, 151]. Histidine is among amino acids which particularly contribute to the formation of amino acid-metal complex. Studies have shown reduction in histidine levels in brain tissues of people with DS [152]. Thus, amino acid abnormalities might increase urinary excretion of Zn and thereby causing Zn deficiency in people with DS. On the other side, since the trace element Se takes part in the formation of some amino acids [153], its deficiency might in turn exacerbate amino acid abnormalities related to the DS and relevant sequelae, for example Zn deficiency. While the uptake of metals in red blood cells appears to be increased as Zn and Cu levels on red blood cells were elevated. Elevation in these metals implies increased activity of CuZn superoxide dismutase enzyme in red blood cells from people with DS [154].

#### Parathyroid hormone (PTH)

It is able to inhibit proximal reabsorption of ions such as Ca and Na [155] and as well to induce salivary secretion of these electrolytes [156]. Therefore elevated salivary levels of Ca and Na might reflect high PTH levels which have been found in individuals with DS [142]. Additionally, high salivary calcium concentrations are suggested as an indicator of osteoporosis [157] and orthopedic problems and low bone mineral density (BMD) are among the most frequently encountered problems in people with DS [158]. Therefore high salivary calcium levels might represent low calcium concentrations in the extracellular fluid, leading to increase in PTH levels and thereby reducing BMD in people with DS. As expected, Ca supplementation could be effective in reducing PTH concentrations and in improving bone turnover in people with DS [159].

#### Other

There are other physiological characteristics related to the DS that may case trace element changes. Of note, many proteins [160] take part in Zn homeostasis [161] and therefore any changes related to these proteins are reflected by altered Zn concentrations. Particularly metallothioneins which are low molecular weight metal-binding proteins have affinity to specific metals (favourably Cu and Zn) and thereby affecting absorption, distribution, and metabolism of these metals [162].

Animal model of DS showed that higher levels of metallothionein 3 in trisomic astrocytes [163] might justify lower free Zn concentrations. The Cu transport is mediated by carriers, importantly the Cu transporter (Ctr) 1. Intestinal malabsorption of Cu induced by deficiency of the Ctr1 gene in murine intestinal cells resulted in tissue copper accumulation [164]. It has to be investigated if intestinal absorption of Cu in people with DS is impaired. Meanwhile the concentrations of superoxide dismutase (SOD1) are elevated in red cells from people with DS [144]. High Cu and SOD1 levels in red cells might lead to oxidative stress and cell degeneration [165]. Because even regular exercise was not helpful in reducing SOD1 levels [166], it seems that effort must be shifted towards preventing Cu/Zn accumulation in red cells. However, regarding Se, the dietary intake is suggested as the best determinant of Se status and Se, with a few exceptions (e.g. parenteral nutrition and the acquired immunodeficiency syndrome), is well-absorbed [167]. Therefore low Se levels in people with DS seem to be the result of inadequate intake. Altogether, at the moment, health professionals must consider assessment of micronutrients (especially Zn) in management of comorbidities and in prevention of potential complications people with DS may develop. When prescribing nutritional supplements, physicians should be aware of all the drugs the patient is taking and be vigilant for the occurrence of toxic effects as well [168, 169].

### Consequences

#### Thyroid dysfunction

People with DS are a vulnerable group to thyroid disorders [170]. Thyroid dysfunction may be a cause of lower intellectual function [171] and lower basal metabolic rate [172]. It is, thus, of importance to identify the causes responsible for this condition. Deficiency of either the trace element Zn [172, 173] or Se [173, 174] can cause impairment in thyroid hormone metabolism. Zn supplementation has proved to be promising in improvement of thyroid function in people with DS and low Zn levels [175, 176]. With regards to Se supplementation, the effect was modest in the general population [177]. It has not been assessed yet whether Se therapy can improve thyroid function in people with DS or not.

#### Immune disorders

Immunodeficiency, infectious diseases, and autoimmune disorders are frequently observed among people with DS [178] to the extent that the DS is expressed as a model of immunodeficiency [179]. Persistent Zn deficiency can lead to inflammation, exacerbating clinical status of patients who suffer from inflammatory and autoimmune diseases [180]. While Se deficiency has been frequently linked with development and exacerbation of viral infections and related complications, e.g. cardiomyopathy [181, 182].

#### Growth abnormalities and orthopedic issues

Ca plays a crucial role in bone growth and in muscle mineralization and its deficiency may cause osteoporosis or osteomalacia [183]. In addition Zn is involved in osteogenic activity and its deficiency might cause or aggravate growth abnormalities in people with DS [184].

### Possible effects of ages and gender

Among studies included in the present meta-analysis, few studies have examined the effect of age [78, 82–84, 185] and/or gender [78, 82, 93, 107] on micronutrient status in people with DS, of which most were related to blood Zn measures. These studies showed that females and males with DS do not differ in Zn levels. However evidence was not conclusive with regards to the effect of age on Zn levels. One study showed that compared with healthy children without DS, children with DS had lower Zn levels, which were closely comparable to those observed in elderly healthy people without DS [84]. Additionally, one study demonstrated that Se concentrations in plasma and erythrocyte tend to increase with age in people with and without DS. They also tend to be higher in women than men [108]. Other studies indicated no effect of age or gender.

### Future directions

A limitation of all such meta-analyses is that they cannot directly clarify the cause and effect. More clearly it has not been answered yet whether micronutrient differences in people with DS are a result of their sedentary behavior and nutritional intake, as DS is a condition associated with decreased nutrient needs and limited exercise capacity [186], or a characteristic of the Syndrome like their different growth pattern. In the former case, longitudinal studies monitoring micronutrient measures and their relationship with clinical status and intake in people with DS may address this issue. The latter case raises a series of fundamental questions based on previous experiences about amino acid abnormalities and thyroid dysfunction which, as mentioned above, are considered a characteristic of DS which normalization by treatment has been proven ineffective in improving their clinical status. Thus fundamental, observational or clinical studies may reveal a. whether the micronutrient profile in DS is correlated with clinical status or QoL, b. whether micronutrient supplements are able to improve these. As it remains a likely possibility that a specific different physiological pattern of amino acids, hormones and micronutrients does reflect DS.

## Supporting information

S1 PRISMA Checklist PRISMA checklist. (DOC)

S1 Text Search strategy. (DOCX)

S1 Table Trace elements and Down syndrome. (DOCX)

S2 Table Minerals and Down syndrome. (DOCX)

S3 Table Vitamins and Down syndrome. (DOCX)

S4 Table Summary of meta-analyses. (DOCX)

S1 Fig PRISMA flow diagram. (DOC)
