# Supplementary material for: Systematic review and meta-analysis shows a specific micronutrient profile in people with Down Syndrome: Lower blood calcium, selenium and zinc, higher red blood cell copper and zinc, and higher salivary calcium and sodium
Source: PLoS One. 2017 Apr 19;12(4):e0175437. doi: 10.1371/journal.pone.0175437 (PMC5396920; doi:10.1371/journal.pone.0175437)
Supplement: S1 Text — (DOCX) [file pone.0175437.s002.docx]

We used the following mesh terms in title, abstract, or key words;

[Down Syndrome OR Down's syndrome OR trisomy 21] AND [vitamin OR vitamins OR provitamin OR pro-vitamin OR thiamine OR riboflavin OR niacin OR panthotenate OR panthotenic acid OR pyridoxine OR pyrodoxal OR pyridoxamine OR biotin OR choline OR methylmalonic acid OR methylmalonate OR folic acid OR folate OR cyanocobalamine OR retinol OR ascorbic acid OR tocopherol OR carotenoid OR carotenoids OR alpha carotene OR alpha-carotene OR beta carotene OR beta-carotene OR cryptoxanthin OR lutein OR lycopene OR zeaxanthin OR nutrient OR nutrients OR nutrition OR nutritional OR diet OR dietary OR diets OR micronutrient OR micronutrients OR mineral OR minerals OR boron OR cobalt OR chromium OR copper OR fluoride OR Chloride OR iodine OR sodium OR potassium OR calcium OR iodine OR Iron OR manganese OR molybdenum OR magnesium OR selenium OR zinc] AND [Subject OR subjects OR human OR humans OR patient OR patients OR inpatient OR inpatients OR individual OR individuals OR person OR persons OR people OR participant OR participants OR volunteer OR volunteers OR case OR cases OR control OR controls OR children OR child OR kid OR kids OR adult OR adults]
