# Supplementary material for: Systematic review and meta-analysis shows a specific micronutrient profile in people with Down Syndrome: Lower blood calcium, selenium and zinc, higher red blood cell copper and zinc, and higher salivary calcium and sodium
Source: PLoS One. 2017 Apr 19;12(4):e0175437. doi: 10.1371/journal.pone.0175437 (PMC5396920; doi:10.1371/journal.pone.0175437)
Supplement: S1 Table — (DOCX) [file pone.0175437.s003.docx]

| **First author**  **Year** | **Element**  **Specimen** | **Study population (N)**  **Male %**  **Age** | **Study levels**  **Mean (SD)** | **Control population (N)**  **Male %**  **Age** | **Control levels**  **Mean (SD)** |
| --- | --- | --- | --- | --- | --- |
| Meguid 2001  Egypt | Selenium Whole blood | Down's Syndrome with trisomy 21 (n = 8)  Range: 9-36 mon | 14.2 (2.2) | Controls (n = 15)  Range: 9-36 mon | 20.4 (2.7) |
|  |  | Down's Syndrome (de novo translocations 21/22 (n = 3) and 21/21 (n = 2)) (n = 5)  Range: 11-33 mon | 13.9 (0.9) |  |  |
|  |  | Down's Syndrome (musaicism) (n = 5)  Range: 12-22 mon | 13.2 (1.2) |  |  |
|  |  | Down's Syndrome (n = 18)  Range : 9-36 mon | 13.84 (1.72) |  |  |
|  | Copper  Plasma | Down's Syndrome with trisomy 21 (n = 8)  Range: 9-36 mon | 2.1 (0.8) | Controls (n = 15)  Range: 9-36 mon | 1.2 (0.2) |
|  |  | Down's Syndrome (de novo translocations 21/22 (n = 3) and 21/21 (n = 2)) (n = 5)  Range: 11-33 mon | 2.8 (0.8) |  |  |
|  |  | Down's Syndrome (musaicism) (n = 5)  Range: 12-22 mon | 2.3 (0.3) |  |  |
|  |  | Down's Syndrome (n = 18)  Range : 9-36 mon | 2.35 (0.76) |  |  |
|  | Zinc  Plasma | Down's Syndrome with trisomy 21 (n = 8)  Range: 9-36 mon | 1.3 (0.6) | Controls (n = 15)  Range: 9-36 mon | 1.2 (0.2) |
|  |  | Down's Syndrome (de novo translocations 21/22 (n = 3) and 21/21 (n = 2)) (n = 5)  Range: 11-33 mon | 1.2 (0.5) |  |  |
|  |  | Down's Syndrome (musaicism) (n = 5)  Range: 12-22 mon | 2.5 (1) |  |  |
|  |  | Down's Syndrome (n = 18)  Range : 9-36 mon | 1.61 (0.9) |  |  |
| Kanavin 2000  Norway | Selenium  Serum | Down’s syndrome (n = 38)  Mean ± SD: 33±11.1 years | 1.2625 (0.1775) | Controls (n = 39) | 1.4 (0.185) |
|  | Zinc  Serum |  | 14.4 (2.8) |  | 15.91 (2) |
|  | Copper  Serum |  | 18.5 (3.2) |  | 18.8 (3.2) |
| Teksen 1998  Turkey | Selenium  Plasma | Down's syndrome (n = 20)  Range: 3-16 years | 113.67 (33.59) | Controls (n = 15) | 124.47 (27.63) |
|  | Copper  Plasma |  | 0.94 (0.17) |  | 1.08 (0.17) |
|  | Zinc  Plasma |  | 1.44 (0.25) |  | 1.46 (0.3) |
| Halsted 1970  USA | Zinc  Plasma | Down's syndrome (n = 10)  Range: 6-13 years | 64 (8) | Healthy (n = 26)  Range: 3-13 years | 89 (13) |
| Fabris 1984  Italy | Zinc  Plasma | Down's syndrome (n = 21)  Range: 3-16 years | 86.3 (18.33) | Normal controls (n = 11)  Range: 3-25 years | 105 (7.9599) |
|  |  |  |  | Normal controls (n = 11)  Range: 65-80 years | 81.9 (11.28) |
| Milunsky 1970  US state | Zinc  Plasma | Down's syndrome (n = 10)  Male %: 60  Mean ± SD: 7.591 ± 2.209557 years | 64 (7.5) | Healthy (n = 16)  Male %: 43.75  Mean ± SD: 8.7575 ± 2.103925 years | 90 (12.4) |
|  | Zinc  Red cell | Down's syndrome (n = 6)  Male %: 33.33  Mean ± SD: 7.168333 ± 2.640912 years | 13.5 (2.4) | Healthy (n = 15)  Male %: 46.67  Mean ± SD: 8.8 ± 2.177154 years | 9.5 (1.4) |
|  | Zinc  Leukocyte | Down's syndrome (n = 4) | 0.01437 (0.0014) | Healthy (n = 5) | 0.00948 (0.00447) |
| Gromadzinska 1988  Poland | Selenium  Whole blood  Erythrocyte  Plasma | Down's syndrome (n = 6)  Range: 6-16 years | 70.2 (12.7) | Controls (n = 77)  Range: 6-16 years | 101.8 (16.2) |
|  |  |  | 116.5 (23.8) | Controls (n = 74)  Range: 6-16 years | 142.2 (31.5) |
|  |  |  | 47.8 (11.9) | Controls (n = 77)  Range: 6-16 years | 76.6 (13.1) |
|  | Selenium  Whole blood  Erythrocyte  Plasma | Down's syndrome (n = 8)  Range: 17-30 years | 68.1 (14.3) | Controls (n = 32)  Range: 17-30 years | 118.1 (16.1) |
|  |  |  | 110.3 (37.5) | Controls (n = 32)  Range: 17-30 years | 167.3 (33.3) |
|  |  |  | 46.4 (11.4) | Controls (n = 32)  Range: 17-30 years | 90.2 (17.5) |
| Kedziora 1986  Poland | Selenium  Erythrocytes | Down's syndrome (n = 14) | 1.6 (0.28) | Healthy (n = 81) | 2.15 (0.46) |
| L. Farzin  2014 | Iran | Down's syndrome (n = 54)  Range: 6-38 years | 87.1 (14.31) | Healthy (n = 60)  Range: 6-40 years | 94.1 (19.47) |
| Neve 1983  France | Selenium  Plasma  Erythrocyte | Down's syndrome (n = 28)  Down's syndrome (n = 28) | 0.9 (0.18)  4.67 (1.12) | Healthy (n = 32)  Healthy (n = 32) | 1.22 (0.27)  4.34 (0.82) |
|  | Zinc  Plasma  Erythrocyte | Down's syndrome (n = 29)  Down's syndrome (n = 28) | 13.5 (3.5)  679 (73) | Healthy (n = 32)  Healthy (n = 31) | 13 (2)  382 (84) |
|  | Copper  Plasma  Erythrocyte | Down's syndrome (n = 26)  Down's syndrome (n = 26) | 23.5 (6.5)  57.1 (8.3) | Healthy (n = 25)  Healthy (n = 25) | 20.8 (5.5)  32.6 (6.1) |
| Meguid 2010  Egypt | Zinc  Serum | Down's syndrome (n = 42)  Mean ± SD: 5 ± 1.1 years | 72.3 (9.6) | Healthy (n = 48)  Mean ± SD: 5.6 ± 2 years | 98.5 (3.08) |
|  | Copper Serum | Down's syndrome (n = 42)  Mean ± SD: 5 ± 1.1 years | 120 (7.5) | Healthy (n = 48)  Mean ± SD: 5.6 ± 2 years | 129 (10.6) |
| Siqueira 2007  US | Zinc  Saliva | Down’s syndrome (n = 20)  Range: 12-60 months | 0.003 (0.0009) | Healthy (n = 18)  Range: 12-60 months | 0.003 (0.001) |
| Marques 2007  Brazil | Zinc  Plasma  Erythrocyte  Urine | Down’s syndrome (n = 30)  Male %: 53.33%  Range: 10-19 years | 67.6 (25.6)  49.2 (8.5)  244.3 (194.9) | Healthy (n = 32)  Male %: 40.63%  Mean: 13.47 years | 68.9 (22.3)  35.9 (6.1)  200.3 (236.4) |
| Garcez 2005  Brazil | Iron  Serum | Down’s syndrome (n = 50)  Male %: 50%  Mean ± SD (range): 14.2 ± 6.62 (3-24) years | 63.64 (27.27) | Control (n = 50)  Male %: 50%  Mean ± SD : 14.2 ± 6.62 years | 59.1 (18.2) |
| Yenigun 2004  Turkey | Zinc  Hair | Down’s syndrome (n = 19)  Range: 2-6 years | 95.18 (56.1) | Typically developing control (n = 11)  Range: 2-6 years | 208.88 (152.37) |
| Siqueira 2004  Brazil | Zinc  Saliva | Down’s syndrome (n = 22)  Male %: 54.54  Range: 6-10 years | 0.002 (0.0009) | Healthy (n = 21)  Male %: 52.38  Range: 6-10 years | 0.002 (0.0012) |
| Torsdottir 2001  Iceland | Copper  Plasma | Down's syndrome (n = 35)  Male %: 77.143%  Mean (range): 37 (18-53) years | 16.1 (12.28) | Healthy (n = 35) | 16.2 (11.6) |
| Kanavin 1988  Norway | Zinc  Serum | Down's syndrome (n = 38)  Male %: 60.5%  Median (range): 32 (16-62) years | 13.725 (3.025) | Mentally retarded patients without DS (n = 39) | 16.25 (1.9) |
| Kadrabova 1995  Slovak Republic | Selenium  Serum | Down’s syndrome (trisomy 21) (n = 16)  Range: 4-23 years | 43.2 (1.7) | Controls (n = 16) | 50.1 (1.6) |
|  | Copper  Serum |  | 1.34 (0.2) |  | 1.1 (0.2) |
|  | Zinc  Serum |  | 0.83 (0.08) |  | 0.96 (0.12) |
|  | Magnesium  Serum |  | 21.7 (1.6) |  | 21 (1.6) |
| Stabile 1991  Italy | Zinc  Serum | Down’s syndrome (n = 38)  Male %: 57.9%  Mean ± SD: 7 ± 4.1 years | 0.67 (0.15) | Healthy (n = 20)  Male %: 55 %  Mean ± SD: 6.9 ± 4.4 years | 1.02 (0.24) |
| Sustrova 1994  Slovakia | Zinc  Serum | Down's syndrome (n = 20)  Range: 1-6 years | 0.75 (0.13416) | Controls (n = 19)  Range: 1-6 years | 1.01 (0.1308) |
|  |  | Down's syndrome (n = 45)  Range: 6-15 years | 0.81 (0.13416) | Controls (n = 20)  Range: 6-15 years | 1.04 (0.18) |
|  |  | Down's syndrome (n = 40)  Range: 15-35 years | 0.92 (0.1265) | Controls (n = 20)  Range: 15-35 years | 1.04 (0.18) |
| David 1996  Italy | Iron  Serum | Down's syndrome (n = 17)  Range: 2-5 years | 72 (32.1) | Healthy (n = 23)  Range: 2-5 years | 71.5 (26.5) |
|  |  | Down's syndrome (n = 23)  Range: 5-10 years | 74 (25.1) | Healthy (n = 25)  Range: 5-10 years | 69.2 (20.2) |
|  |  | Down's syndrome (n = 10)  Range: 10-15 years | 84.2 (29.4) | Healthy (n = 20)  Range: 10-15 years | 89 (23.8) |
| Franceschi 1988  Italy | Zinc  Plasma | Down's syndrome (n = 18)  Male %: 61.1%  Mean ± SD: 7 ± 0.83 years | 85.8 (4.5)  71.3 (3.4) | Healthy (n = 15)  Male %: 60% | 105.2 (2.4) |
|  | Copper  Plasma |  | 74.8 (3.8)  67.9 (2.3) |  | 71.4 (3.4) |
| Noble 1988  USA | Zinc  Plasma | Down's syndrome (n = 11) | 1.5 (0.1) | Healthy (n = 11) | 1.27 (0.05) |
| Toledo 1997  France | Zinc  Serum | Down's syndrome (n = 105)  Male %: 47.6%  Range: 3mon-20 years | 14.89 (1.65) | Control (n = 105) | 19.04 (1.74) |
| Anneren 1985  Sweden | Selenium  Plasma | Down's syndrome (n = 65)  Male %: 50.8%  Range: 1 mon-56 years | 100 (17.3) | Healthy (n = 90)  Male %: 58.9%  Range: 2 mon-54 years | 87.9 (20.2) |
|  | Selenium  Erythrocyte |  | 100 (17.3) |  | 87.9 (20.2) |
| Anneren 1985  Sweden | Copper  Erythrocyte | Down’s syndrome (n = 11)  Male %: 72.73%  Range: 4-10 years | 3.4 (0.6) | Healthy (n = 13)  Male %: 46.2%  Range: 5-14 years | 1.1 (0.3) |
|  | Zinc  Erythrocyte |  | 8.5 (7.4) |  | 17 (4.4) |
|  | Fe  Erythrocyte |  | 320 (125.9) |  | 560 (296.3) |
|  | Manganese  Erythrocyte |  | 1 (0.15) |  | 1.4 (0.4) |
| Neve 1984  Belgium | Zinc  Plasma  RBC | Down’s syndrome (n = 10)  Range: 21-51 years | 11.9 (2)  691 (72) | Healthy (n = 25)  Range: 23-57 years | 15 (3.3) |
|  |  | Down’s syndrome (n = 10)  Range: 23-52 years | 12.7 (1.5)  687 (73) | Healthy (n = 7)  Range: 24-49 years | 12.7 (1.8)  549 (70) |
|  | Copper  Plasma  RBC | Down’s syndrome (n = 10)  Range: 21-51 years | 17 (4.4)  53.2 (4.7) | Healthy (n = 25)  Range: 23-57 years | 19.7 (3.6)  40.1 (4.6) |
|  |  | Down’s syndrome (n = 10)  Range: 23-52 years | 17.1 (4.2)  51.3 (10.4) | Healthy (n = 7)  Range: 24-49 years | 18.2 (5)  39.5 (3.5) |
|  | Selenium  Plasma  RBC | Down’s syndrome (n = 10)  Range: 21-51 years | 1.06 (0.11)  5.76 (0.82) | Healthy (n = 25)  Range: 23-57 years | 1.25 (0.23)  6.28 (0.89) |
|  |  | Down’s syndrome (n = 10)  Range: 23-52 years | 0.89 (0.09)  4.48 (0.94) | Healthy (n = 7)  Range: 24-49 years | 1.03 (0.2)  5.53 (0.66) |
| McBean 1974  USA | Zinc  Serum | Down’s syndrome (n = 9)  Male %: 44.44  Mean ± SD: 34 ± 8 years | 98 (16) | Controls (n = 20)  Male %: 50  Mean ± SD: 32 ± 10.198 years | 101 (15) |
| Cutress 1972  New Zealand | Zinc  Manganese  Copper  Fe  Whole saliva | Down’s syndrome (n = 31)  Range: 6-22 years | 0.8 (0.4)  0.02 (0.02)  0.36 (0.18)  0.61 (0.81) | Non-trisomy mentally retarded (n = 28)  Male %: 57.14  Range: 6-22 years | 0.6 (0.6)  0.02 (0.01)  0.18 (0.11)  0.6 (1.06) |
|  | Zinc  Manganese  Copper  Fe  Parotid saliva | Down’s syndrome (n = 16)  Range: 7-22 years | 0.2 (0.3)  0.01 (0.01)  0.31 (0.17)  0.07 (0.03) | Non-trisomy mentally retarded (n = 16)  Male %: 50  Range: 8-23 years | 0.6 (0.6)  0.02 (0.01)  0.48 (0.17)  0.15 (0.03) |
| Fernández 2005  Venezuela | Zinc  Plasma | Down's syndrome (n = 35)  Range: 6mon-6 years | 1839 (361) | Healthy (n = 35)  Range: 6mon-6 years | 1374 (867) |
|  | Copper  Plasma |  | 805 (261) |  | 767 (288) |
| Soto-Quintana 2003  Venezuela | Zinc  Plasma | Down's syndrome (n = 43)  Male %: 51.16%  Mean ± SD: 2.3 ± 2 years | 809 (228.9) | Healthy (n = 40)  Male %: 50%  Mean ± SD: 2.3 ± 2 years | 1034.6 (166.9) |
| Licastro 1992  Italy | Zinc  Plasma | Down's syndrome (n = 25)  Male %: 76  Range: 6-15 years | 76 (3) | Normal controls (n = 14)  Male %: 64.3%  Range: 9-13 years | 97 (4) |
| Barlow 1981  England | Zinc  Copper  Iron  Manganese  Hair | Down’s syndrome (n = 69)  Male %: 100  Mean ± SD: 39.4 ± 9.3 years | 114.4 (30.8)  8.5 (3.1)  11.9 (8.3)  0.42 (0.35) | Normal controls (n = 35)  Male %: 100  Mean ± SD: 32.7 ± 20.1 years | 120.3 (27.2)  24.2 (12.3)  24.4 (12.8)  2.67 (1.76) |
|  |  |  |  | Patients without Down's syndrome (n = 49)  Male %: 100  Mean ± SD: 41.4 ± 8.3 years | 102.3 (13.9)  12.8 (3.6)  12.8 (11.5)  0.73 (1.25) |
|  |  | Down’s syndrome (n = 67)  Male %: 0  Mean ± SD: 37.1 ± 12.7 years | 122.5 (24.4)  13.4 (6.4)  15.4 (11.2)  0.21 (0.24) | Normal controls (n = 51)  Male %: 0  Mean ± SD: 39.9 ± 22.9 years | 141.2 (36.3)  35.5 (34.6)  20.3 (10.2)  2.1 (1.52) |
|  |  |  |  | Patients without Down's syndrome (n = 20)  Male %: 0  Mean ± SD: 53.7 ± 16.6 years | 134.4 (36.7)  11.9 (2.5)  3.7 (1.8)  0.44 (0.46) |
