# Supplementary material for: Systematic review and meta-analysis shows a specific micronutrient profile in people with Down Syndrome: Lower blood calcium, selenium and zinc, higher red blood cell copper and zinc, and higher salivary calcium and sodium
Source: PLoS One. 2017 Apr 19;12(4):e0175437. doi: 10.1371/journal.pone.0175437 (PMC5396920; doi:10.1371/journal.pone.0175437)
Supplement: S2 Table — (DOCX) [file pone.0175437.s004.docx]

| **Study (YOP)**  **Location** | **Mineral Specimen** | **Study population** | **Study levels**  **mean (SD)** | **Control population** | **Control levels mean (SD)** |
| --- | --- | --- | --- | --- | --- |
| Schwertner 2016  Brazil | Calcium  Saliva | Down's syndrome (n = 61)  Male %: 57.40%  Mean ± SD: 9 ± 2.06 years | 31.91 (1.67405) | Control (n = 83)  Male %: 54.2%  Mean ± SD: 9.43 ± 1.84 years | 29.283 (6.948) |
|  | Phosphorus Saliva |  | 0.287 (0.12593) |  | 0.27 (0.0963) |
| Stagi 2015  Italy | Calcium  Plasma | Down's syndrome (n = 31)  Male %, 54.84%  Range: 4.5 - 18.9 years | 2.42 (0.14) | Healthy (n = 99)  Male %: 51.51%  Range: 4.8 - 19.8 years | 2.51 (0.1) |
|  | Phosphate  Plasma |  | 1.32 (0.24) |  | 1.3 (0.32) |
| Areias 2013  Portugal | Calcium  Saliva | Down's syndrome (n = 45)  Male %: 49%  Mean ± SD: 12.7 ± 4 years | 3.34 (2.27) | Control (n = 45)  Male %: 60%  Mean ± SD: 12.8 ± 3.7 years | 4.26 (3.13) |
|  | Phosphate  Saliva |  | 11 (5.7) |  | 12.8 (6) |
|  | Potassium  Saliva |  | 15.8 (7.6) |  | 17.1 (6.7) |
|  | Chloride  Saliva |  | 20.5 (5.1) |  | 22.3 (4,6) |
| Yamato 2009  Japan | Calcium  Serum | Down’s syndrome (n = 27)  Male %: 59.26%  Mean ± SD: 8.6 ± 4.6 years | 8.2 (0.9) | Healthy (n = 14)  Male %: 57.14%  Mean ± SD: 12 ± 3.9 years | 9.2 (0.2) |
| Siqueira 2007  US | Sodium  Saliva | Down’s syndrome (n = 20)  Range: 12-60 months | 8.7 (0.87) | Healthy (n = 18)  Range: 12-60 months | 5.44 (1.19) |
|  | Potassium  Saliva |  | 5.6 (1.04) |  | 7.93 (1.49) |
|  | Phosphorus  Saliva |  | 1.09 (0.12) |  | 1.17 (0.17) |
|  | Calcium  Saliva |  | 0.5 (0.08) |  | 0.47 (0.1) |
|  | Magnesium  Saliva |  | 0.06 (0.03) |  | 0.06 (0.02) |
| Sakadamis 2002  Greece | Calcium  Serum | Down's syndrome (n = 11)  Male %: 100%  Mean ± SD: 26.45 ± 3.91 years | 2.43 (0.21) | Healthy (n = 12)  Mean ± SD: 24.92 ± 3.06 years | 2.5 (0.01) |
|  | Phosphate  Serum |  | 1.47 (0.2) |  | 1.48 (0.18) |
| Siqueira 2004  Brazil | Sodium  Saliva | Down’s syndrome (n = 22)  Male %: 54.54  Range: 6-10 years | 9.49 (2.1) | Healthy (n = 21)  Male %: 52.38  Range: 6-10 years | 5.69 (1.54) |
|  | Potassium  Saliva |  | 5.32 (1.88) |  | 7.52 (1.28) |
|  | Phosphorus  Saliva |  | 1.16 (0.31) |  | 1.1 (0.31) |
|  | Calcium  Saliva |  | 0.45 (0.16) |  | 0.42 (0.09) |
|  | Magnesium  Saliva |  | 0.06 (0.014) |  | 0.06 (0.027) |
| Hestnes 1991  Norway | Sodium  Serum | Down's syndrome (n = 29)  Male %: 79.3  Mean ± SD: 43.6 ± 11.8 years | 142 (4) | Control (n = 29)  Male %: 79.3  Mean ± SD: 44.6 ± 12.3 years | 140 (3) |
|  | Potassium  Serum |  | 4.1 (0.3) |  | 4 (0.4) |
|  | Calcium  Serum |  | 2.3 (0.09) |  | 2.3 (0.1) |
|  | Phosphate  Serum |  | 1.1 (0.14) |  | 1.1 (0.15) |
|  | Chloride  Serum |  | 102 (3) |  | 102 (3) |
| Jara 1991  Chile | Chloride  Saliva | Down’s syndrome (n = 19)  Male %: 52.6%  Mean (range): 19.5 (10-25) years | 19.69 (8.04) | Normal (n = 20)  Male %: 85 %  Mean (range): 14 (10-19) years | 19.3 (3.49) |
|  |  |  |  | Non-Down mentally retarded (n = 21)  Male %: 80.95 %  Mean (range): 15 (11-22) years | 19.25 (10.6) |
|  |  | Down’s syndrome (n = 20) | 16.5 (8.6) | Normal (n = 10) | 23.93 (11.82) |
|  |  |  |  | Non-Down mentally retarded (n = 13) | 16.4 (5.8) |
|  | Sodium  Saliva | Down’s syndrome (n = 19)  Male %: 52.6%  Mean (range): 19.5 (10-25) years | 22.79 (11.35) | Normal (n = 20)  Male %: 85 %  Mean (range): 14 (10-19) years | 17.1 (3.07) |
|  |  |  |  | Non-Down mentally retarded (n = 21)  Male %: 80.95 %  Mean (range): 15 (11-22) years | 19 (13.65) |
|  |  | Down’s syndrome (n = 20) | 22.6 (17.8) | Normal (n = 10) | 20.5 (19.91) |
|  |  |  |  | Non-Down mentally retarded (n = 13) | 21.1 (11.9) |
|  | Potassium  Saliva | Down’s syndrome (n = 19)  Male %: 52.6%  Mean (range): 19.5 (10-25) years | 21.01 (2.21) | Normal (n = 20)  Male %: 85 %  Mean (range): 14 (10-19) years | 23.58 (2.93) |
|  |  |  |  | Non-Down mentally retarded (n = 21)  Male %: 80.95 %  Mean (range): 15 (11-22) years | 23.27 (3.98) |
|  |  | Down’s syndrome (n = 20) | 20 (4.9) | Normal (n = 10) | 19 (2.6) |
|  |  |  |  | Non-Down mentally retarded (n = 13) | 20.21 (3.31) |
| Chapman 1967  Surrey | Sodium  Parotid saliva | Down’s syndrome (n = 33)  Mean (range): 6 (1-12) years | 26.1 (21.05) | Normal controls (n = 31)  Range: 18-43 years | 5.9 (3) |
|  |  |  |  | Severely subnormal control subjects (n = 29)  Mean (range): 6 (2-14) years | 10.4 (9.375) |
| Barlow 1981  England | Calcium  Magnesium  Hair | Down’s syndrome (n = 69)  Male %: 100  Mean ± SD: 39.4 ± 9.3 years | 391 (167)  34.1 (18.8) | Normal controls (n = 35)  Male %: 100  Mean ± SD: 32.7 ± 20.1 years | 726 (472)  63.8 (31.6) |
|  |  |  |  | Patients without Down's syndrome (n = 49)  Male %: 100  Mean ± SD: 41.4 ± 8.3 years | 583 (241)  37.3 (15.5) |
|  |  | Down’s syndrome (n = 67)  Male %: 0  Mean ± SD: 37.1 ± 12.7 years | 525 (290)  41.1 (26) | Normal controls (n = 51)  Male %: 0  Mean ± SD: 39.9 ± 22.9 years | 1123 (686)  81.2 (62.1) |
|  |  |  |  | Patients without Down's syndrome (n = 20)  Male %: 0  Mean ± SD: 53.7 ± 16.6 years | 1031 (567)  46.3 (32.1) |
| Cutress 1972  New Zealand | Calcium  Magnesium  Whole saliva | Down’s syndrome (n = 36)  Male %: 52.78  Range: 6-22 years | 47.4 (11.4)  4.7 (1.9) | Non-trisomy mentally retarded (n = 28)  Male %: 57.14  Range: 6-22 years | 38.3 (11.7)  3.6 (2.6) |
|  | Calcium  Magnesium  Parotid saliva | Down’s syndrome (n = 20)  Male %: 40  Range: 7-22 years | 21 (5)  2 (0.9) | Non-trisomy mentally retarded (n = 16)  Male %: 50  Range: 8-23 years | 18.9 (8)  2 (0.71) |
|  | Sodium  Potassium  Whole saliva | Down’s syndrome (n = 36)  Male %: 52.78  Range: 6-22 years | 178 (79)  815 (154) | Non-trisomy mentally retarded (n = 28)  Male %: 57.14  Range: 6-22 years | 208 (122)  698 (90) |
|  | Sodium  Potassium Parotid saliva | Down’s syndrome (n = 20)  Male %: 40  Range: 7-22 years | 257 (202)  742 (119) | Non-trisomy mentally retarded (n = 16)  Male %: 50  Range: 8-23 years | 125 (86)  828 (179) |
|  | Phosphate  Whole saliva | Down’s syndrome (n = 36)  Male %: 52.78  Range: 6-22 years | 148 (50) | Non-trisomy mentally retarded (n = 28)  Male %: 57.14  Range: 6-22 years | 118 (33) |
|  | Phosphate  Parotid saliva | Down’s syndrome (n = 20)  Male %: 40  Range: 7-22 years | 135 (22) | Non-trisomy mentally retarded (n = 16)  Male %: 50  Range: 8-23 years | 128 (57) |
