# Supplementary material for: Systematic review and meta-analysis shows a specific micronutrient profile in people with Down Syndrome: Lower blood calcium, selenium and zinc, higher red blood cell copper and zinc, and higher salivary calcium and sodium
Source: PLoS One. 2017 Apr 19;12(4):e0175437. doi: 10.1371/journal.pone.0175437 (PMC5396920; doi:10.1371/journal.pone.0175437)
Supplement: S3 Table — (DOCX) [file pone.0175437.s005.docx]

| **Study (YOP)**  **Location** | **Vitamin** | **Study population** | **Study levels**  **mean (SD)** | **Control population** | **Control levels mean (SD)** |
| --- | --- | --- | --- | --- | --- |
| Parisotto 2015  Brazil | Vitamin E  Plasma | Down's syndrome (n = 21)  Mean ± SD: 7.7 ± 3.18 years | 4.9 (1.65) | Healthy (n = 20)  Mean ± SD: 6.7 ± 3 years | 4.48 (3.48) |
| Sukla 2015  India | Vitamin B12  Plasma | Down's syndrome (n = 151)  Male %: 73.68%  Median: around 2 years | 190.67 (50.37) | Healthy (n = 200)  Male %: NM  Age: Newborns (majority) and less than 10 years (minority) | 216.67 (59.26) |
|  | Folate Plasma |  | 3.23 (0.815) |  | 4.23 (1.78) |
| Stagi 2015  Italy | 25(OH)D Plasma | Down's syndrome (n = 31)  Male %: 54.84%  Range: 4.5 - 18.9 years | 14.34 (8.31) | Healthy (n = 99)  Male %: 51.51%  Range: 4.8 - 19.8 years | 27.04 (7.47) |
| de Sousa 2015  Brazil | Vitamin C Saliva | Down's syndrome (n = 30)  Range: 14-24 years | 10.94 (4.47) | Healthy (n = 30) | 8.58 (5.02) |
| Real de Asua 2014  Spain | Vitamin D Blood | Down's syndrome (n = 48)  Male %: 64.58%  Mean ± SD: 36 ± 11 years | 33.6 (20.8) | Healthy (n = 33)  Male %: 60.61%  Mean ± SD: 42.5 ± 12.5 years | 27.8 (11.4) |
| Obermann-Borst 2011  Netherlands | Folate  Serum  RBC | Down's syndrome (n = 8) | 45.225 (24.3153)  1476.25 (99.18) | Healthy (n = 186)  Male %: 55.9%  Median (range): 17.3 (13-24.9) years | 41.25 (19.254)  1158.5 (344.487) |
|  | Vitamin B12 Serum | Down's syndrome (n = 8) | 424.75 (21.9232) | Healthy (n = 186)  Male %: 55.9%  Median (range): 17.3 (13-24.9) years | 582.25 (145.092) |
| Meguid 2010  Egypt | Folate  Serum | Down's syndrome (n = 42)  Mean ± SD: 5 ± 1.1 years | 7.03 (3.12) | Healthy (n = 48)  Mean ± SD: 5.6 ± 2 years | 11.4 (4.07) |
|  | Vitamin B12 Serum | Down's syndrome (n = 42)  Mean ± SD: 5 ± 1.1 years | 942 (604) | Healthy (n = 48)  Mean ± SD: 5.6 ± 2 years | 1090 (691) |
|  | Vitamin E Serum | Down's syndrome (n = 42)  Mean ± SD: 5 ± 1.1 years | 1.59 (0.14) | Healthy (n = 48)  Mean ± SD: 5.6 ± 2 years | 1.7 (0.14) |
|  | Vitamin C Serum | Down's syndrome (n = 42)  Mean ± SD: 5 ± 1.1 years | 0.21 (0.02) | Healthy (n = 48)  Mean ± SD: 5.6 ± 2 years | 0.29 (0.05) |
| Chavez 2010  Venezuela | Retinol Serum | Down's syndrome (n = 16)  Male %: 62.5%  Mean ± SD (range): 8.2 ± 4.1 (2-16) years | 33.6 (13.4) | Karyotypically normal (n = 50)  Male %: 46%  Mean ± SD (range): 10.4 ± 3.7 (2-16) years | 35.8 (9.9) |
| Licastro 2006  Italy | Vitamin B12  Serum | Down's syndrome (n = 13)  Male %: 100%  Mean: 60 years | 278 (99) | Healthy (n = 20)  Male %: 100%  Mean: 60 years | 396 (120) |
|  | Folate  Serum |  | 9.3 (5.1) |  | 16.9 (5.8) |
| Ercis 1996  Turkey | Vitamin A  Serum | Down's syndrome (n = 71)  Male %: 58%  Mean (range): 2.833 (0.1667-25) years | 32.54 (12.25) | Control (n = 41)  Male %: 56%  Mean (range): 3.1667 (0.1667-17) years | 36.68 (10.65) |
| Pueschel 1990  USA | Vitamin A  Serum | Down's syndrome (n = 33)  Male %: 45.5%  Mean (range): 18 (6-28) years | 106 (87.9) | Intellectually normal persons (n = 14)  Male %: 50%  Mean (range): 20.9 (12-33) years | 136.5 (70.3) |
|  | Carotene  Serum |  | 136.8 (56.8) |  | 147 (35.4) |
| Fillon-Emery 2004  France | Folate  RBC | Down's syndrome (n = 160)  Male %: 54.38%  Mean ± SD (range): 26 ± 4 (15-46) years | 4684.66 (2833.505) | Healthy (n = 160)  Range: 15-46 years | 1028.4 (456.633) |
|  | Folate  Serum |  | 167.514 (180.1) |  | 13.4 (7.5895) |
|  | Vitamin B12  Serum |  | 419.1 (160.1255) |  | 372.5 (121.4315) |
| Bras 1989  Portugal | Vitamin C  Plasma | Down's syndrome (n = 5)  Range: 9mon-22 years | 10.1 (1.1) | Healthy (n = 6) | 10.4 (4.5) |
|  | Vitamin E  Plasma | Down's syndrome (n = 9)  Range: 9mon-22 years | 12.7 (2.5) | Healthy (n = 7) | 11.9 (1.5) |
| David 1996  Italy | Folate  Serum | Down's syndrome (n = 17)  Range: 2-5 years | 5.9 (1.5) | Healthy (n = 23)  Range: 2-5 years | 6.3 (1.2) |
|  |  | Down's syndrome (n = 23)  Range: 5-10 years | 6.4 (1.9) | Healthy (n = 25)  Range: 5-10 years | 5.3 (1.4) |
|  |  | Down's syndrome (n = 10)  Range: 10-15 years | 4.9 (1.6) | Healthy (n = 20)  Range: 10-15 years | 5.3 (1.5) |
|  | Folate  RBC | Down's syndrome (n = 17)  Range: 2-5 years | 233.3 (69.4) | Healthy (n = 23)  Range: 2-5 years | 227.8 (66.3) |
|  |  | Down's syndrome (n = 23)  Range: 5-10 years | 219.4 (41.3) | Healthy (n = 25)  Range: 5-10 years | 238.2 (78.6) |
|  |  | Down's syndrome (n = 10)  Range: 10-15 years | 184.7 (47.6) | Healthy (n = 20)  Range: 10-15 years | 226.4 (47.6) |
|  | Vitamin B12  Serum | Down's syndrome (n = 17)  Range: 2-5 years | 593.8 (219.4) | Healthy (n = 23)  Range: 2-5 years | 508.9 (192.9) |
|  |  | Down's syndrome (n = 23)  Range: 5-10 years | 562.2 (158.3) | Healthy (n = 25)  Range: 5-10 years | 523.5 (144.7) |
|  |  | Down's syndrome (n = 10)  Range: 10-15 years | 506.3 (203.7) | Healthy (n = 20)  Range: 10-15 years | 386.9 (96.9) |
| Hestnes 1991  Norway | Vitamin B12  Serum | Down's syndrome (n = 29)  Male %: 79.3  Mean ± SD: 43.6 ± 11.8 years | 357 (145) | Control (n = 29)  Male %: 79.3  Mean ± SD: 44.6 ± 12.3 years | 475 (160) |
| Coburn 1983  US state | Vitamin B6 (Pyridoxal)  Serum | Down's syndrome (n = 4)  Male %: 100%  Mean ± SD: 25 ± 4 years | 19 (4) | Nonspecific diagnosis (n = 9)  Male %: 66.7%  Mean ± SD: 24 ± 4 years | 21 (1) |
|  |  | Down's syndrome (n = 5)  Male %: 80%  Mean ± SD: 25 ± 4 years | 24 (4) | Nonspecific diagnosis (n = 9)  Male %: 77.8%  Mean ± SD: 23 ± 4 years | 23 (8) |
|  | Vitamin B6 (Pyridoxal phosphate)  Serum | Down's syndrome (n = 4)  Male %: 100%  Mean ± SD: 25 ± 4 years | 60 (27) | Nonspecific diagnosis (n = 9)  Male %: 66.7%  Mean ± SD: 24 ± 4 years | 61 (27) |
|  |  | Down's syndrome (n = 5)  Male %: 80%  Mean ± SD: 25 ± 4 years | 64 (13) | Nonspecific diagnosis (n = 9)  Male %: 77.8%  Mean ± SD: 23 ± 4 years | 58 (27) |
|  | Vitamin B6 (Pyridoxic acid)  Serum | Down's syndrome (n = 4)  Male %: 100%  Mean ± SD: 25 ± 4 years | 35 (30) | Nonspecific diagnosis (n = 9)  Male %: 66.7%  Mean ± SD: 24 ± 4 years | 50 (18) |
|  |  | Down's syndrome (n = 5)  Male %: 80%  Mean ± SD: 25 ± 4 years | 47 (19) | Nonspecific diagnosis (n = 9)  Male %: 77.8%  Mean ± SD: 23 ± 4 years | 43 (16) |
| Barden 1977  USA | Vitamin A  Serum | Down's syndrome (n = 44)  Male %: 50%  Mean ± SD: 15.5 ± 6.1 years | 39.3 (9.8) | Non-Down's syndrome (n = 56)  Male %: 50%  Mean ± SD: 14.8 ± 6.4 years | 34.7 (9.4) |
|  |  |  |  | Normal (n = 40)  Male %: 62.5%  Mean ± SD: 14.1 ± 7.9 years | 38.9 (10.6) |
|  | Carotene  Serum | Down's syndrome (n = 44)  Male %: 50%  Mean ± SD: 15.5 ± 6.1 years | 172.8 (75.6) | Non-Down's syndrome (n = 56)  Male %: 50%  Mean ± SD: 14.8 ± 6.4 years | 196.7 (70.5) |
|  |  |  |  | Normal (n = 40)  Male %: 62.5%  Mean ± SD: 14.1 ± 7.9 years | 99.4 (29.8) |
| Barden 1978  USA | Vitamin A  Serum | Down's syndrome (n = 29)  Male %: 44.8% | 39.74 (8.77) | Non-Down's syndrome (n = 56)  Male %: 48.9% | 34.4 (9.8) |
|  | Carotene  Serum |  | 187.5 (80.2) |  | 198.4 (68.2) |
| Nandha Kumar 2014  India | Folic acid  Plasma | Down's syndrome (n = 108)  Male %: 59.3%  Mean ± SD: 3.03 ± 4.1 years | 8.12 (2.42) | Healthy (n = 110) | 8.48 (2.24) |
| Parisotto 2014  Brazil | Vitamin E  Plasma | Down's syndrome (n = 21)  Male %: 57.14%  Mean ± SD: 7.7 ± 3.18 years | 4.894 (0.1064) | Healthy (n = 18)  Male %: 55.56%  Mean ± SD: 6.7 ± 3 years | 4.468 (0.21) |
| Garlet 2013  Brazil | Vitamin E  Plasma | Down's syndrome (n = 20)  Mean ± SD: 7.7 ± 3.18 years | 4.84 (2.13) | Healthy (n = 18)  Male %: 55.56%  Mean ± SD: 6.7 ± 3 years | 4.44 (2.36) |
| Del Arco 1992  Spain | 25(OH)D-March  Serum | Down's syndrome (n = 21)  Male %: 47.6 %  Range: 3-10 years | 20.2 (5.6) | Children with disorders not affecting their general health status (n = 12) | 18.7 (8.5) |
|  | 25(OH)D-October  Serum |  | 32.9 (8.1) |  | 31.8 (6) |
|  | 1,25(OH)2D-March  Serum |  | 47.7 (8) |  | 50 (16.9) |
|  | 1,25(OH)2D-October  Serum |  | 48.1 (11.1) |  | 47.2 (7.5) |
|  | 24,25(OH)2D-March  Serum |  | 1.4 (0.6) |  | 1 (0.4) |
|  | 24,25(OH)2D-October  Serum |  | 1.5 (0.5) |  | 2.7 (0.6) |
| Shah 1989  US state | Retinol  Plasma | Down's syndrome (n = 12)  Male %: 66.67 %  Range: 22-58 years | 29.4 (8.08) | Normals (n = 12)  Male %: 50 %  Range: 24-62 years | 40.4 (10.8) |
|  | Beta-carotene  Plasma |  | 39 (11.2) |  | 18 (7.7) |
|  | Alpha-tocopherol  Plasma  Erythrocyte |  | 0.57 (0.1)  1.36 (0.4) |  | 0.95 (0.3)  2.2 (0.8) |
| Howell 1973  England | Folate  Serum  Red cell | Down's syndrome (n = 113)  Male %: 59.3 | 5.7 (3.9)  244.15 (112.56) | Controls (n = 106)  Male %: 58.49 | 5.02 (4.23)  254.8 (120.1) |
|  | Vitamin B12  Serum |  | 356.7 (168.9) |  | 429.4 (211.5) |
| Pallardo 2006  Italy | Vitamin C  Plasma | Down's syndrome (n = 13)  Age: < 15 years old | 32.2 (13.2) | Healthy (n = 19)  Age: < 15 years old | 20.3 (10) |
|  |  | Down's syndrome (n = 5)  Age: > 15 years old | 27.4 (21.7) | Healthy (n = 9)  Age: > 15 years old | 24.2 (9.6) |
