# Supplementary material for: Systematic review and meta-analysis shows a specific micronutrient profile in people with Down Syndrome: Lower blood calcium, selenium and zinc, higher red blood cell copper and zinc, and higher salivary calcium and sodium
Source: PLoS One. 2017 Apr 19;12(4):e0175437. doi: 10.1371/journal.pone.0175437 (PMC5396920; doi:10.1371/journal.pone.0175437)
Supplement: S4 Table — (DOCX) [file pone.0175437.s006.docx]

| **Outcome** | **Comparisons (n)** | **Cases (n) Controls (n)** | **Heterogeneity chi^2^ *p* value** | **Inconsistency I^2^ %** | **Effect measure SMD 95% CI** | **Overall effect Z**  ***p* value** | **References** |
| --- | --- | --- | --- | --- | --- | --- | --- |
| Blood Zn | 30 | 794/768 | 405.87 (<.00001) | 93 | $-$1.30 [$-$1.75, $-$0.84] | 5.59 (<.00001) | (75, 76, 78, 79, 81-83, 85-87, 89-98, 101, 102, 104, 105) |
| Plasma Zn | 17 | 343/356 | 234.06 (0.0005) | 93 | $-$1.23 [$-$1.93, $-$0.54] | 3.47 (0.0005) | (75, 76, 81-83, 85, 87, 89-91, 95, 97, 98, 101, 102) |
| Serum Zn | 13 | 451/412 | 107.29 (<.00001) | 89 | $-$1.44 [$-$1.93, $-$0.95] | 5.75 (<.00001) | (79, 86, 88, 92-94, 96, 104, 105) |
| RBC Zn | 6 | 95/123 | 69.70  (<.00001) | 93 | 1.88 [0.48, 3.28] | 2.63 (0.009) | (76, 81, 83, 84, 102) |
| RBC Zn | 4 | 78/95 | 15.95 (0.001) | 81 | 2.62 [1.59, 3.66] | 4.97 (<.00001) | (81, 83, 102) |
| Hair Zn | 3 | 155/97 | 4.58 (0.1) | 56 | $-$0.54 [$-$0.97, $-$0.12] | 2.50 (0.01) | (80, 100) |
| Saliva Zn | 4 | 89/83 | 6.38 (0.09) | 53 | $-$0.05 [$-$0.49, 0.40] | 0.21 (0.84) | (77, 99, 103) |
| Blood Cu | 14 | 348/390 | 72.82 (<.00001) | 82 | 0.01 [$-$0.36, 0.37] | 0.03 (0.98) | (81, 83, 85, 93, 95-97, 101, 104, 105, 128, 142) |
| Plasma Cu | 8 | 172/172 | 35.52 (<.00001) | 80 | 0.29 [$-$0.31, 0.72] | 0.77 (0.44) | (81, 83, 85, 95, 97, 101, 142) |
| Serum Cu | 6 | 176/218 | 25.93 (<.0001) | 81 | $-$0.24 [$-$0.72, 0.24] | 0.94 (0.35) | (93, 96, 104, 105, 128) |
| RBC Cu | 5 | 83/125 | 13.88 (0.008) | 71 | 2.77 [1.96, 3.57] | 6.74 (<.00001) | (81, 83, 84, 128) |
| Blood Se | 16 | 287/517 | 162.31 (<.00001) | 91 | $-$0.99 [$-$1.55, $-$0.43] | 3.44 (0.0006) | (81, 83, 93, 95-97, 105, 106, 122) |
| Plasma Se | 9 | 147/278 | 102.21 (<.00001) | 92 | $-$0.66 [$-$1.49, 0.18] | 1.53 (0.12) | (81, 83, 95, 106, 122) |
| Serum Se | 4 | 108/115 | 5.35 (0.15) | 44 | $-$0.60 [$-$0.97, $-$0.23] | 3.21 (0.001) | (93, 96, 105) |
| Whole blood Se | 3 | 32/124 | 3.22 (0.20) | 38 | $-$2.60 [$-$3.32, $-$1.89] | 7.11 (<.00001) | (97, 122) |
| RBC Se | 9 | 141/341 | 72.25 (<.00001) | 89 | $-$0.30 [$-$0.97, 0.37] | 0.87 (0.38) | (81, 83, 106, 122, 126) |
| Serum Fe | 4 | 100/118 | 0.94 (0.82) | 0 | 0.12 [$-$0.15, 0.39] | 0.86 (0.39) | (114, 120) |
| Blood Ca | 4 | 98/154 | 9.92 (0.02) | 70 | $-$0.63 [$-$1.16, $-$0.09] | 2.28 (0.02) | (123, 137, 140, 143) |
| Serum Ca | 3 | 67/55 | 8.33 (0.02) | 76 | $-$0.56 [$-$1.36, 0.24] | 1.38 (0.17) | (123, 137, 143) |
| Saliva Ca | 8 | 274/243 | 104.04 (<.00001) | 93 | 0.85 [0.38, 1.33] | 3.53 (0.0004) | (77, 99, 103, 107, 115, 138) |
| Saliva Cl | 3 | 115/100 | 15.78 (0.001) | 81 | $-$0.03 [$-$0.69, 0.63] | 0.08 (0.94) | (107, 115, 125) |
| Saliva Mg | 4 | 98/83 | 2.37 (0.50) | 0 | 0.17 [$-$0.13, 0.46] | 1.12 (0.26) | (77, 99, 103) |
| Blood P | 3 | 71/140 | 0.08 (0.96) | 0 | 0.03 [$-$0.27, 0.32] | 0.19 (0.85) | (123, 137, 140) |
| Saliva P | 6 | 204/211 | 12.71 (0.03) | 61 | 0.07 [$-$0.25, 0.40] | 0.44 (0.66) | (77, 99, 103, 107, 138) |
| Saliva K | 7 | 182/158 | 50.45 (<.00001) | 88 | $-$0.51 [$-$1.18, 0.16] | 1.50 (0.13) | (77, 99, 103, 107, 125) |
| Saliva Na | 8 | 203/173 | 56.99 (<.00001) | 88 | 1.04 [0.39, 1.69] | 3.14 (0.002) | (77, 99, 103, 111, 125) |
| Blood vitamin D | 4 | 121/156 | 44.50 (<.00001) | 93 | $-$0.25 [$-$1.33, 0.82] | 0.46 (0.64) | (117, 136, 140) |
| Serum vitamin A | 9 | 215/192 | 19.68 (0.01) | 59 | 0.07 [$-$0.28, 0.42] | 0.37 (0.71) | (108, 109, 112, 118, 135) |
| Serum vitamin B9 | 11 | 510/898 | 69.31 (<.00001) | 86 | -0.20 [-0.54 – 0.14] | 1.17 (0.24) | (104, 114, 119, 124, 127, 129, 130, 141) |
| RBC vitamin B9 | 7 | 196/520 | 18.84 (0.004) | 68 | 0.05 [-0.30 – 0.39] | 0.25 (0.80) | (114, 119, 124, 130) |
| Blood vitamin B12 | 11 | 431/817 | 37.02 (<.0001) | 73 | $-$0.25 [$-$0.52, 0.02] | 1.83 (0.07) | (104, 114, 119, 123, 124, 127, 130, 141) |
| Blood vitamin E | 6 | 125/121 | 57.58 (<.00001) | 91 | 0.12 [$-$0.84, 1.09] | 0.25 (0.80) | (104, 110, 121, 132, 133, 139) |
| Plasma vitamin E | 5 | 83/73 | 42.18 (<.00001) | 91 | 0.32 [$-$0.83, 1.47] | 0.55 (0.58) | (110, 121, 132, 133, 139) |
