# Supplementary material for: Systematic review and meta-analysis shows a specific micronutrient profile in people with Down Syndrome: Lower blood calcium, selenium and zinc, higher red blood cell copper and zinc, and higher salivary calcium and sodium
Source: PLoS One. 2017 Apr 19;12(4):e0175437. doi: 10.1371/journal.pone.0175437 (PMC5396920; doi:10.1371/journal.pone.0175437)
Supplement: S1 Fig — (DOC) [file pone.0175437.s007.doc]

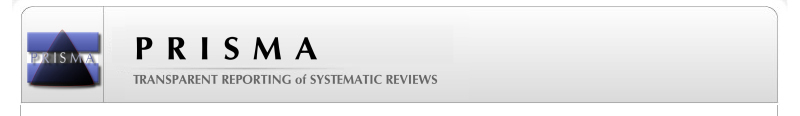
**PRISMA 2009 Flow Diagram**

**Screening**

**Included**

**Eligibility**

**Identification**

Records identified through database searching
(n = 4656)

Records after duplicates removed
(n = 3206)

Records screened
(n = 2505)

Records excluded
(n = 2388)

Full-text articles assessed for eligibility
(n = 117)

Full-text articles excluded, with reasons
(n = 48)

Studies included in qualitative synthesis
(n = 69)

Studies included in quantitative synthesis (meta-analysis)
(n = 69)
